# Osteolytic bone destruction resulting from relapse of a testicular tumour 23 years after inguinal orchiectomy and adjuvant chemotherapy: a case report

**DOI:** 10.4076/1752-1947-3-8702

**Published:** 2009-07-31

**Authors:** Christos Kalaitzis, Athanasios Bantis, Georgios Tsakaldimis, Stylianos Giannakopoulos, Efthimios Sivridis, Stavros Touloupidis

**Affiliations:** 1 Department of Urology, Democritus University of Thrace, Alexandroupolis, Greece

## Abstract

**Introduction:**

Late relapse of a testicular germ cell tumour is an uncommon occurrence. We report a case of osteolytic bone metastasis appearing 23 years after the initial treatment of a metastatic testicular mixed tumour (choriocarcinoma and embryonal carcinoma). This is one of the longest periods of recurrence reported for testicular germ cell tumours.

**Case presentation:**

A 52-year-old Caucasian man who underwent a right inguinal orchiectomy due to testicular tumour in 1984 presented to our outpatient clinic in a generally bad condition of health and with severe pain of his right hip joint and os ischii caused by osteolytic metastasis.

**Conclusions:**

This case emphasizes the need for a life-long follow-up of patients with primary metastatic testicular cancer.

## Introduction

The prognosis of malignant testicular tumour has dramatically improved due to cisplatin-based chemotherapy. When diagnosed at an early stage, the disease has a cure rate of 95% to 100%. In advanced stages, cure rates of up to 70% are still possible [1]. However, after surgical therapy and cisplatin containing chemotherapy, up to 10% of patients monitored have developed relapses despite being pronounced tumour-free. The majority of these relapses developed during the first two years [2]. However, in a small number of patients, late relapses were noted [2].

The most complete report of late relapse was published by Baniel et al. [3]. The authors demonstrated that germ cell tumours in a situation of late relapse exhibit a biological behaviour that is different from the primary tumours. An early relapse incidence of 2% to 3% was calculated. The median time of appearance for late relapse was six years, and the main localisation was the retroperitoneum and the lung, following an increase in the level of the tumour marker alpha-fetoprotein (AFP). Late relapse was sensitive to chemotherapy, although chemotherapy alone is normally insufficient to achieve a complete cure. The most efficacious therapy was surgical resection of the tumour mass.

We report here a case of a late relapse of a germ cell tumour, which occurred 23 years after orchiectomy and following four cycles of chemotherapy and five years of complete remission.

## Case presentation

A 52-year-old man presented to our outpatient clinic in a bad general condition of health and with severe pain of his right hip joint and os oschii. The pain developed approximately three months prior to presentation.

The patient's medical history revealed a right inguinal orchiectomy for the removal of testicular tumour performed in France in 1984. The rest of his anamnesis was clinically insignificant.

In 1984, the patient's histopathology revealed a mixed tumour, a choriocarcinoma and an embryonic carcinoma, with retroperitoneal and pulmonary metastases. To treat this, an orchiectomy was performed, followed by four cycles of bleomycin, etoposide and cisplatin (BEP) chemotherapy. Follow-up assessments were initially performed at an interval of three months, and then at an interval of six months until the fifth postoperative year.

When the patient presented to our clinic, he appeared to be in bad general health, with severe pain in his right hip joint. His serum level of alpha-fetoprotein was 169.48 ng/ml (normal range 0.0 to 7.0 ng/ml) and beta-human chorionic gonadotropin (ß-hCG) was 1.30 mIU/ml (normal range <5.0 mIU/ml). The patient's lactate dehydrogenase (LDH) level was 1155 U/L (normal range 230 to 460 U/L) and his alkaline phosphatase level was 104 U/L (normal range is 3 to 128 U/L).

**Figure 1:**
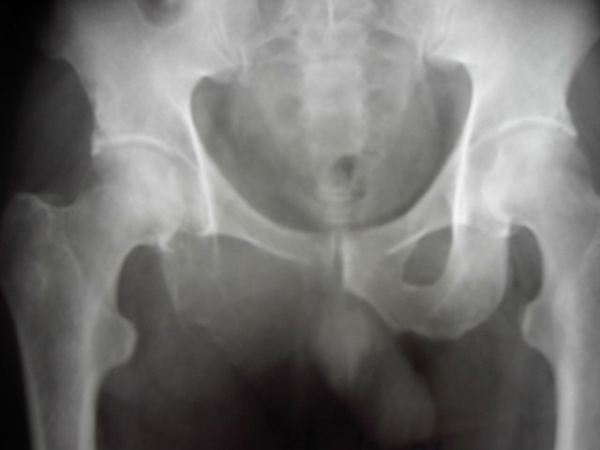
**Osteolytic destruction of the patient's right os ischii**.

**Figure 2:**
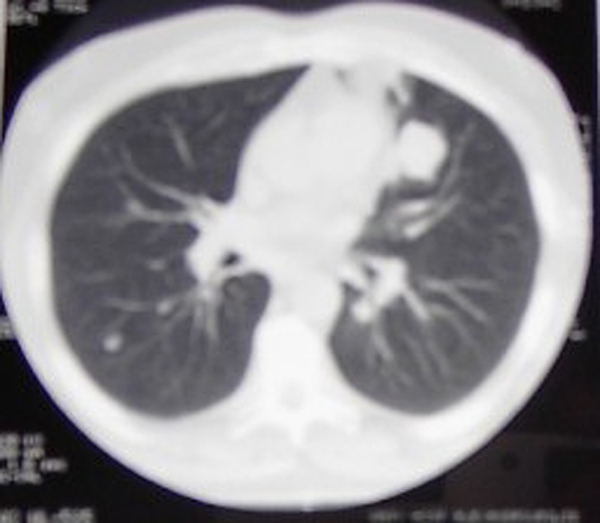
**CT scan of the chest shows a tumour mass in the left lung**.

Radiological investigation, which was later confirmed through computer tomograms, demonstrated that there was an osteolytic destruction of the patient's os ischii and hip joint (Figure 1). In addition, enlarged lymphatic nodes were found on the patient's retroperitoneal space. Two pulmonary metastases were also found in his left lung (Figure 2).

**Figure 3:**
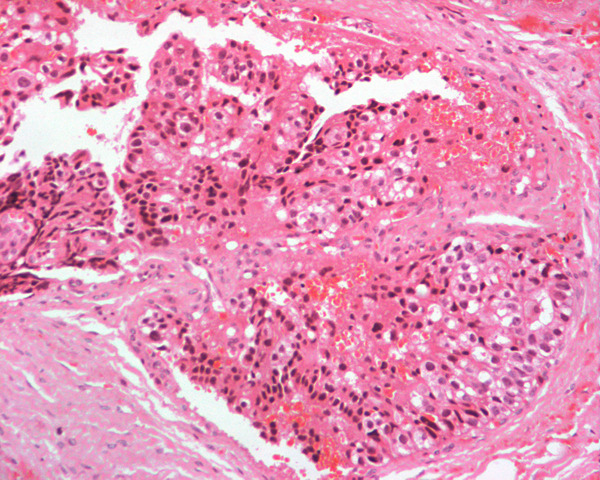
**Bone biopsy with a focus of metastatic germ cells with abundant clear or weakly eosinophilic cytoplasm (Haematoxylin and eosin)**.

A biopsy of the lytic bone lesion was carried out to confirm the diagnosis. Histological and immunohistochemical investigation of the biopsy cylinders showed an anaplastic seminoma (Figure 3). Immunohistochemical testing was positive for alpha-fetoprotein but negative for hCG, carcinoembryonic antigen (CEA), CD30 and CD117 (C kit).

Due to strong pain, analgesia with peripheral and central analgesics was initiated. During the next three days, however, the state of our patient dramatically declined and he died a day before the results of the biopsy were known.

## Discussion

After initial therapy, patients with germ cell tumours who have two years of complete remission have a high cure rate. The probability of a late relapse is between 1.3% and 7% [4][[Bibr B5]]-[6]. Late relapses of nonseminomatous tumours are more frequent [4].

Late relapse is defined as a germ cell tumour that appears more than two years after therapy and complete remission [3]. Of the 81 patients treated at the University of Indiana for late relapse of germ cell tumours, 60% showed late relapse more than five years after primary therapy and complete remission [3].

A series of 1263 patients with germ cell testicular tumour treated at the Department of Radiotherapy and Oncology in Surrey, United Kingdom showed that only 14 patients had late relapse between the 5th and 10th year after primary treatment with a calculated annual risk of 1%. In two patients, late relapse occurred later than 10 years. One of these patients presented with metastatic seminoma, while the other presented with nonseminoma during the clinical stage I, which was then followed up by a wait-and-see attitude [7].

The possible mechanisms for the development of late relapses are identified as: 1) the presence of mature teratoma in the germ cell tumour; 2) the growth of the remaining tumour not destroyed by the chemotherapy; and 3) the development of a secondary germ cell carcinoma and the microscopic persistence of tumour cells with atypical biological behaviour [8].

Albers et al. wrote the European Association of Urology (EAU) guidelines on testicular cancer in a limited update in March 2009. Precise recommendations for minimum follow-up schedules in advanced nonseminomatous germ cell tumours and seminoma were provided, indicating the procedures that should be performed at one, two and three to five years after initial therapy and thereafter [9].

Late relapses respond weakly to chemotherapy. Surgical excision remains the best treatment. Baniel et al. showed that chemotherapy alone yields poor results in patients with late relapse [3]. Tumour-free status was achieved by chemotherapy in only two out of 81 patients. In patients who did not initially receive chemotherapy to treat the germ cell tumour, complete remission could be reached during late relapse by chemotherapy alone [3].

## Conclusions

Late relapse of malignant germ cell tumours are a rare occurrence. Until recently, it was generally considered sufficient to perform follow-up assessments for up to five years after initial therapy. However, metastatic nonseminomatous tumours form an exception to this general rule, and late tumour relapses do occur. It thus seems necessary that follow-up examinations be performed for longer than five years after the primary therapy. This regime is recommended in the latest 2009 update of the EAU guidelines on testicular cancer as written by Albers and his co-authors.

## Abbreviations

AFP: alpha-fetoprotein; BEP: bleomycin, etoposide and cisplatin; CEA: carcinoembryonic antigen; EAU: European Association of Urology; hCG: Human chorionic gonadotropin; LDH: lactate dehydrogenase.

## Consent

We were unable to obtain consent for the publication of this case due to the death of the patient and despite repeated attempts we were unable to trace the next of kin. Every effort has been made to keep the patient's identity anonymous. We would not expect the patient's family to object to publication.

## Competing interests

The authors declare that they have no competing interests.

## Authors' contribution

GS and TG performed the bone biopsy. SE performed the histological examination. BA was a major contributor in writing the manuscript. All authors read and approved the final manuscript.
